# Family history of cancer and gastroesophageal disorders and risk of esophageal and gastric adenocarcinomas: a case–control study

**DOI:** 10.1186/1471-2407-14-60

**Published:** 2014-02-04

**Authors:** Xuejuan Jiang, Chiu-Chen Tseng, Leslie Bernstein, Anna H Wu

**Affiliations:** 1 Department of Ophthalmology and Visual Sciences, University of Illinois at Chicago, Chicago, IL, USA; 2 Department of Preventive Medicine, Keck School of Medicine, University of Southern California/Norris Comprehensive Cancer Center, Los Angeles, CA, USA; 3 Department of Population Sciences, Beckman Research Institute and City of Hope Comprehensive Cancer Center, Duarte, CA, USA; 4 University of Southern California/Norris Comprehensive Cancer Center, 1441 Eastlake Avenue, Los Angeles, CA 90089-9175, USA

**Keywords:** Hiatal hernia, Family history, Esophageal adenocarcinoma, Gastric cardia, Distal gastric cancer

## Abstract

**Background:**

There is a paucity of data on familial risk of developing esophageal adenocarcinoma, gastric cardia adenocarcinoma and distal gastric adenocarcinoma from population-based studies.

**Methods:**

A population-based case–control study of newly diagnosed gastroesophageal adenocarcinoma was conducted in Los Angeles County. This analysis included data of case-patients whom we were able to interview directly (147 patients with esophageal adenocarcinoma, 182 with gastric cardia adenocarcinoma, and 285 with distal gastric adenocarcinoma) and 1,309 control participants. Multivariate polytomous logistic regression was used to estimate odds ratios (ORs) and corresponding 95% confidence intervals (CIs) for the three cancer types.

**Results:**

Risk of esophageal adenocarcinoma was positively associated with a family history of prostate cancer (OR = 2.84; 95% CI = 1.50-5.36) and a family history of hiatal hernia (OR = 2.04; 95% CI = 1.12-3.71). Risk of gastric cardia adenocarcinoma was strongly associated with a family history of esophageal cancer (OR = 5.18; 95% CI = 1.23-21.79) and a family history of hiatal hernia (OR = 2.31; 95% CI = 1.37-3.91). Risk of distal gastric adenocarcinoma was positively associated with a family history of gastric cancer (OR = 2.15; 95% CI = 1.18-3.91), particularly early-onset (before age 50) gastric cancer (OR = 2.82; 95% CI = 1.11-7.15).

**Conclusions:**

This study provides evidence that family history of hiatal hernia is a risk factor for esophageal adenocarcinoma and gastric cardia adenocarcinoma and that cancer in specific sites is associated with risk of esophageal adenocarcinoma, gastric cardia adenocarcinoma, and distal gastric adenocarcinoma. It is important to determine the extent to which shared environmental and genetic factors explain these familial associations.

## Background

Esophageal and gastric cancers are one of the most common cancers in the world, with an estimated 482,300 new esophageal cancer cases and 989,600 new gastric cancer cases diagnosed in 2008 worldwide. However, incidence rates at these two cancer sites vary substantially internationally [1]. In the last few decades, despite the decline in esophageal squamous cell carcinoma and distal gastric cancer in most parts of the world [2], incidence rates of adenocarcinomas of the esophagus (EA) and gastric cardia (GCA) have been rising rapidly in the Western countries, possibly due to the increasing prevalence of two risk factors, obesity and reflux conditions [3,4]. A pooled analysis of individual participant data from 12 epidemiological studies worldwide found increasing risk of both EA and GCA with increasing body mass index (BMI) and evidence for a synergistic interaction between obesity and gastroesophageal reflux (GERD) symptoms [3]. Compared with individuals with a BMI <25 kg/m^2^, individuals with BMI ≥40 kg/m^2^ had a >4 fold increase in EA risk and >3 fold increase in GCA. There are also some reports of stable or declining incidence of GCA in more recent years. A 2012 study [5] reported that in the Netherlands, the incidence for GCA decreased in males but remained stable in females, changes that are unlikely caused by improved disease diagnosis or reclassification. In the United States, incidence of EA is now higher than that of esophageal squamous cell carcinoma [6]. Most EAs are believed to originate from Barrett’s esophagus [7], which is strongly associated with gastroesophageal reflux disease and presence of hiatal hernia. The geographic variations and temporal changes in the incidence of esophageal and gastric cancer indicate an important role of environmental factors in the development of these diseases. There is also evidence implicating an etiologic role of genetic factors. Studies have shown higher risk of esophageal and gastric cancers among close relatives of patients with these diseases [4,8-14]. However, to our knowledge, only one [12] of these three studies [10-12] that have evaluated the familial risk of histology- and site-specific subtypes of esophageal and gastric cancer was a population-based study. For the present analyses, we utilized data from a well-characterized population-based case–control study to assess whether family history of gastrointestinal cancers, other cancers and gastroesophageal disorders (hiatal hernia, any ulcer, gastritis, and Barrett’s esophagus) are associated with risk of EA, GCA, and distal gastric adenocarcinoma (DGA). Our investigation not only separated gastroesophageal adenocarcinoma by anatomical sites, but also investigated the effect of family history of cancer as well as the effect of family history of non-malignant gastroesophageal disorders.

## Methods

### Study population

The details of the study population and design have been described previously [15-20]. Briefly, case patients eligible for this study were men and women between the age of 30 and 74 years newly diagnosed with histologically confirmed, incident EA (International Classification of Disease for Oncology code [ICD-O] C15.0-C15.9), GCA (ICD-O code C16.0), or DCA (ICD-O codes C16.1-C16.6 and C16.8-C16.9) diagnosed between 1992 and 1997. They were identified by the Los Angeles County Cancer Surveillance Program, the population-based cancer registry covering Los Angeles County, a member of the National Cancer Institute’s Surveillance, Epidemiology, and End Results (SEER) program, and the statewide California Cancer Registry. Control participants were subjects without a diagnosis of gastric or esophageal cancer. They were individually matched to each case patient on sex, race, age (±5 years) and neighborhood of residence. A systematic algorithm based on the address of each case patient was used to recruit the case’s matched control [17]. To increase the study’s statistical power, we sought two control participants for each case patient whenever possible.

The study was approved by the Institutional Review Board of the Keck School of Medicine of the University of Southern California and all study procedures adhered to the recommendations of the Declaration of Helsinki. Written informed consent was obtained from each study participant before interview.

In-person structured interviews were conducted with participants. Next-of-kin (NOK) were interviewed when case patients were unable to be interviewed due to death or illness. Although it was not feasible to blind interviewers to case (or NOK) or control status, interviewers and study participants were not aware of the study hypotheses. A total of 947 case patients were interviewed, representing 77% of the 1230 eligible patients who were approached (77% for EA, 74% for GA, and 78% for DGA). Among them, 528 were matched to one control participant, 382 were matched to two or more control participants, and 37 had no eligible control participant identified. For the current analysis, data from 271 NOK case-patient interviews (66 EA, 85 GCA and 120 DGA) were excluded to reduce misclassification of family history. We also excluded 62 case patients and 47 control participants because of extreme caloric intake (so the analysis cohort could be as comparable as possible to those in previous publications from the same study) or missing information on key covariates (smoking, body size and others). A total of 614 case patients (147 with EA, 182 with GCA and 285 with DGA) and 1,309 control participants were included in the statistical analyses.

### Data collection

Cases and their matching controls were interviewed by the same interviewer in almost all instances. A reference date was defined as one year before the date of diagnosis of the case patient; this same reference date was used for each case patient’s matched control subject(s). A structured questionnaire designed specifically for this study was administered during the in-person interview, obtaining data up to the reference date. The interview queried demographic information, smoking history, lifetime use of all types of alcoholic beverages, usual diet, weight at ages 20 and 40 years and on the reference date (referred to as current weight), and height. To assess a participant’s medical history of a list of diseases, we asked if the participant had any of the conditions diagnosed by a physician before the reference date. Conditions of the upper gastrointestinal tract that were asked included gastric ulcer, duodenal ulcer, gastritis, hiatal or diaphragmatic hernia, esophagitis, Barrett’s esophagus, gastresophageal reflux disease, excess acid, and gastric hyperacidity.

In addition, we asked detailed questions regarding history of conditions of esophagus and gastrointestinal tract and history of any cancer among participants’ first-degree relatives. Specifically, each participant was asked about the vital status of his/her natural mother and father, the number of full-brothers and full-sisters, and if any of these immediate family members was ever diagnosed by a physician for gastritis, hiatal hernia, Barrett’s esophagus, any type of ulcer or cancer. If the response was yes for any of the non-malignant conditions, participants were then asked at what age was the relative first diagnosed for the condition. Age at diagnosis was unknown for 15 participants’ father, 13 participants’ mother, and 17 participants’ siblings. If the response was yes for cancer, participants were further asked about the cancer site and age at cancer diagnosis. Cancer sites were coded according to the International Classification of Diseases 9^th^ version (ICD-9) code: any gastrointestinal (ICD-9: 150–159), esophageal (ICD-9: 150), gastric (ICD-9: 151), colorectal (ICD-9: 153–154), hepatic (ICD-9: 155), pancreas (ICD-9: 157), lung (ICD-9: 162), breast (ICD-9: 174), bone/skin/connective tissue (ICD-9: 170–173), oral/upper respiratory organ (ICD-9: 140–149, 160–165), prostate (ICD-9: 185), female reproductive organ (ICD-9: 179–184), lymphatic/hematopoietic (ICD-9: 200, 208) cancer, and cancer of unknown primary site (ICD-9: 199). Numbers of other cancers reported in these relatives were insufficient for reliable analyses. If the participant did not have any siblings, only the parents’ history was counted.

### Statistical analysis

A participant was classified as having a family history of a condition if they reported at least one first-degree relative (biological parent or sibling) with the condition and as having a family history of an early-onset condition if they reported at least one first-degree relative with the condition who was diagnosed before age 50 years. A family history of cancer with unknown age at diagnosis was treated as a family history of late-onset cancer, given that most cancers are diagnosed after age 50. Results were essentially unchanged with or without including subjects with a family history of cancer of unknown age at diagnosis.

Odds ratios (OR) and corresponding 95% confidence intervals (CI) were estimated for associations of family history with risk of EA, GCA, and DGA. To maximize our statistical power, we report results from polytomous logistic regression with adjustment for the matching variables [19] including age (≤49, 50–59, 60–69, ≥70 years), sex (male/female) and race (non-Hispanic white, African American, Hispanic, Asian). We previously showed [19] that this approach provided more precise estimates of the ORs while the magnitude of the estimated ORs was consistent with those obtained in separate conditional logistic regression analyses that preserved the original case–control match within each cancer site. Given that the causal factors for esophageal adenocarcinoma is not entirely known, we chose to adjust for all common risk factors that were suspected to be associated with both gastroesophageal adenocarcinomas and family history of cancer or gastroesophageal disorders: birth place (US born, non-US born), level of education (<high school, high school, some college, college graduate or higher), cigarette smoking status (never, former, and current smoker), body mass index (BMI) at reference age (in quartiles: ≤23, >23-25, >25- ≤ 28, >28 in males, and ≤22, >22-25, >25- ≤ 28.25, >28.25 in females), and history of diabetes were also included as covariates in the analyses. BMI was categorized using sex-specific quartiles rather than the World Health Organization classification to avoid sparse data for some categories. Personal history of other malignancies was also included as a covariate when analyzing the effect of family history of cancer. Additional adjustment for fiber intake had minimal effect on the summary estimates so it was not included in the final model.

Significance of the interaction between family and personal history of hiatal hernia was evaluated using one degree of freedom likelihood ratio test of a product term between the two variables. We conducted these analyses separately for each type of cancer using unconditional logistic regression.

All reported P values are two-sided. All statistical analyses were performed using the SAS 9.2 statistical software (SAS Institute Inc., Cary, NC).

## Results

Demographic characteristics of the cases and controls have been described in detail previously [19]. Control subjects who reported a family history of any cancers were more likely to be older, US born, and non-Hispanic white than those without a family history of any cancer, but those with and without a family history did not differ with regard to gender, level of education, cigarette smoking status, BMI, history of diabetes or reflux symptoms (Table 1). In contrast, control subjects who reported a family history of gastroesophageal disorders (hiatal hernia, any ulcer, gastritis, and Barrett’s esophagus) were more likely to be younger, female, better educated, and have a personal history of reflux symptoms than controls without a family history of gastroesophageal disorders, but were not different in race/ethnicity, place of birth, cigarette smoking status, BMI and history of diabetes.

**Table 1:** Family history of cancer and gastroesophageal disorders by demographic and lifestyle characteristics among control subjects

	**Family history of any cancer**			**Family history of gastroesophageal disorders**		
**N (row percent)**	**No**	**Yes**	***P***^***a***^	**No**	**Yes**	***P***^**a**^
Age			0.005			<0.001
≤49	161 (58.8%)	113 (41.2%)		169 (61.7%)	105 (38.3%)	
50-59	174 (52.6%)	157 (47.4%)		217 (65.8%)	113 (34.2%)	
60-69	215 (48.3%)	230 (51.7%)		337 (76.8%)	102 (23.2%)	
≥70	118 (47.8%)	129 (52.2%)		196 (79.7%)	50 (20.3%)	
Gender			0.70			0.003
Male	499 (51.8%)	464 (48.2%)		703 (73.6%)	252 (26.4%)	
Female	169 (50.6%)	165 (49.4%)		216 (64.7%)	118 (35.3%)	
Race			*<0.001*			0.19
Non-Hispanic white	382 (46.8%)	434 (53.2%)		590 (72.6%)	223 (27.4%)	
African American	44 (51.2%)	42 (48.8%)		64 (76.2%)	20 (23.8%)	
Hispanics	174 (61.7%)	108 (38.3%)		187 (66.6%)	94 (33.4%)	
Asian American	68 (60.2%)	45 (39.8%)		78 (70.3%)	33 (29.7%)	
Country of birth			*<0.001*			0.67
U.S.	463 (47.4%)	513 (52.6%)		689 (71.0%)	282 (29.0%)	
Other countries	205 (63.9%)	116 (36.1%)		230 (72.3%)	88 (27.7%)	
Education			0.28			0.016
<High school	134 (58.8%)	94 (41.2%)		170 (75.6%)	55 (24.4%)	
High school	116 (47.5%)	128 (52.5%)		181 (74.8%)	61 (25.2%)	
Some college	184 (49.1%)	191 (50.9%)		263 (70.5%)	110 (29.5%)	
College graduate or higher	234 (52.0%)	216 (48.0%)		305 (67.9%)	144 (32.1%)	
Cigarette smoking status			0.70			0.74
Never smokers	269 (52.2%)	246 (47.8%)		362 (70.8%)	149 (29.2%)	
Former smokers	291 (51.0%)	279 (49.0%)		412 (72.8%)	154 (27.2%)	
Current smokers	108 (50.9%)	104 (49.1%)		145 (68.4%)	67 (31.6%)	
Body mass index (BMI)^b^			0.60			0.60
Quartile1	207 (57.8%)	171 (45.2%)		266 (70.9%)	109 (29.1%)	
Quartile 2	158 (48.0%)	171 (52.0%)		236 (72.2%)	91 (27.8%)	
Quartile 3	150 (50.3%)	148 (49.7%)		219 (74.0%)	77 (26.0%)	
Quartile 4	153 (52.4%)	139 (47.6%)		198 (68.0%)	93 (32.0%)	
Diabetes			0.42			0.91
No	616 (51.8%)	572 (48.2%)		841 (71.2%)	340 (28.8%)	
Yes	52 (47.7%)	57 (52.3%)		78 (72.2%)	30 (27.8%)	
Personal history of reflux symptoms			0.33			0.026
No	582 (52.1%)	536 (47.9%)		804 (72.4%)	306 (27.6%)	
Yes	86 (48.0%)	93 (52.0%)		115 (64.2%)	64 (35.8%)	

^a^P values were estimated using Fisher’s exact test for binary variables, Mantel-Haenszel Chi-Square test for ordinal multi-level variables, and Chi-square test for nominal multi-level variables.

^b^Quartile cut points for current BMI are ≤23, >23-25, >25- ≤ 28, >28 kg/m^2^ for males, and ≤22, >22-25, >25- ≤ 28.25, >28.25 kg/m^2^ for females.

Table 2 presents the risk of the three cancer types in relation to family history of cancer among first degree relatives, with adjustment for matching variables (age, sex, and race) as well as other risk factors of these adenocarcinoma identified in our previous investigations. Results adjusted for matching factors only are also presented in Additional file 1: Table S1 for comparison. Family history of cancer was unknown for 17 participants (5 cases and 12 controls). There was essentially no subject with partially unknown family history of cancer. Risk of EA was positively associated with a family history of prostate cancer (OR = 2.84; 95% CI = 1.50-5.36). Risk of EA was also inversely associated with family history of breast cancer (OR = 0.60; 95% CI = 0.28-1.28) although the 95% CI for this OR estimate did not exclude 1.0. Risk of GCA was positively associated with a family history of esophageal cancer (OR = 5.18; 95% CI = 1.23-21.79) but was not associated with family history of other gastrointestinal cancers. There was no association between family history of any non-gastrointestinal cancer and GCA risk. Risk of DGA was positively associated with a family history of any gastrointestinal cancers (OR = 1.45; 95% CI = 0.95-2.23) although the 95% CI for this OR estimate did not exclude 1.0. This increase in risk seemed to be more pronounced among those with a family history of early-onset (before age 50 years) gastrointestinal cancer (OR = 1.84; 95% CI = 0.90-3.78) than among those whose family members had later-onset of their gastrointestinal cancers (OR = 1.31; 95% CI = 0.80-2.16). The P for trend estimated from Cochran-Armitage trend test over the three ordered groups: “no family history”, “having a family history of late-onset gastrointestinal cancer”, and “having a family history of early-onset gastrointestinal cancer” was 0.057. When family history of gastrointestinal cancers was analyzed separately by tumor site, risk of DGA was increased among individuals with a family history of gastric cancer (OR = 2.15; 95% CI = 1.18-3.91), particularly early-onset gastric cancer (OR = 2.82; 95% CI = 1.11-7.15; not shown in the tables). Risk of DGA was also increased among individuals with a family history of pancreatic cancer (OR = 2.17; 95% CI = 0.72-6.51), even though the association was not statistically significant. Further adjustments for personal history of hiatal hernia, reflux symptoms, and sibship size did not substantially change these associations for EA, GCA, and DGA (data not shown).

**Table 2:** Family history of cancer and risk of esophageal and gastric adenocarcinoma

**History of cancer among first degree relatives**		**EA**			**GCA**			**DGA**		
	**Controls**	**Cases**	**OR (95% CI)**^**a**^	***P***^***a***^	**Cases**	**OR (95% CI)**^**a**^	***P***^***a***^	**Cases**	**OR (95% CI)**^**a**^	***P***^***a***^
Any cancer										
No	668	68	1.00 (ref)		90	1.00 (ref)		160	1.00 (ref)	
Yes	629	78	1.02 (0.68-1.54)	*0.92*	91	1.04 (0.71-1.53)	*0.83*	122	0.83 (0.60-1.15)	*0.27*
Late-onset	481	58	0.96 (0.62-1.49)	*0.85*	63	0.96 (0.63-1.46)	*0.84*	89	0.81 (0.56-1.16)	*0.25*
Early-onset	148	20	1.24 (0.66-2.35)	*0.50*	28	1.33 (0.74-2.38)	*0.34*	33	0.90 (0.54-1.48)	*0.67*
***Gastrointestinal cancer***										
Any gastrointestinal cancer^b^										
No	1101	125	1.00 (ref)		148	1.00 (ref)		226	1.00 (ref)	
Yes	196	21	0.87 (0.48-1.59)	*0.66*	33	1.19 (0.71-1.97)	*0.51*	56	1.45 (0.95-2.23)	*0.087*
Late-onset	153	16	0.85 (0.44-1.65)	*0.63*	26	1.23 (0.70-2.14)	*0.47*	40	1.31 (0.80-2.16)	*0.28*
Early-onset	43	5	0.98 (0.29-3.33)	*0.97*	7	1.04 (0.36-3.02)	*0.95*	16	1.84 (0.90-3.78)	*0.095*
Esophageal cancer										
No	1290	145	1.00 (ref)		176	1.00 (ref)		280	1.00 (ref)	
Yes	7	1	-	*-*	5	5.18 (1.23-21.79)	*0.025*	2	-	*-*
Gastric cancer										
No	1241	141	1.00 (ref)		171	1.00 (ref)		260	1.00 (ref)	
Yes	56	5	0.92 (0.32-2.65)	*0.87*	10	1.57 (0.72-3.45)	*0.26*	22	2.15 (1.18-3.91)	*0.012*
Colorectal cancer										
No	1222	138	1.00 (ref)		171	1.00 (ref)		260	1.00 (ref)	
Yes	75	8	0.94 (0.39-2.27)	*0.89*	10	0.70 (0.27-1.79)	*0.46*	22	1.21 (0.61-2.39)	*0.59*
Liver cancer										
No	1267	139	1.00 (ref)		179	1.00 (ref)		276	1.00 (ref)	
Yes	30	7	1.94 (0.64-5.83)	*0.24*	2	-	*-*	6	0.51 (0.13-2.01)	*0.34*
Pancreatic cancer										
No	1275	145	1.00 (ref)		176	1.00 (ref)		276	1.00 (ref)	
Yes	22	1	-	*-*	5	1.55 (0.44-5.43)	*0.49*	6	2.17 (0.72-6.51)	*0.17*
***Non-gastrointestinal cancer***										
Lung cancer										
No	1202	132	1.00 (ref)		164	1.00 (ref)		267	1.00 (ref)	
Yes	95	14	1.03 (0.48-2.24)	*0.93*	17	0.92 (0.43-1.98)	*0.84*	15	1.05 (0.52-2.11)	*0.90*
Upper respiratory organ cancer										
No	1172	128	1.00 (ref)		157	1.00 (ref)		260	1.00 (ref)	
Yes	125	18	0.98 (0.50-1.93)	*0.96*	24	1.22 (0.67-2.20)	*0.51*	22	1.02 (0.57-1.85)	*0.94*
Skin/bone/connective tissue cancer										
No	1225	141	1.00 (ref)		174	1.00 (ref)		272	1.00 (ref)	
Yes	72	5	0.61 (0.21-1.74)	*0.35*	7	0.44 (0.14-1.46)	*0.18*	10	1.06 (0.40-2.79)	*0.90*
Lymphatic/hematopoietic cancer										
No	1256	141	1.00 (ref)		179	1.00 (ref)		272	1.00 (ref)	
Yes	41	5	1.49 (0.56-3.96)	*0.43*	2	-	*-*	10	0.90 (0.32-2.55)	*0.84*
Prostate cancer										
No	1224	130	1.00 (ref)		170	1.00 (ref)		272	1.00 (ref)	
Yes	73	16	2.84 (1.50-5.36)	*0.001*	11	1.45 (0.70-3.01)	*0.32*	10	0.44 (0.17-1.16)	*0.10*
Breast cancer										
No	1168	137	1.00 (ref)		167	1.00 (ref)		258	1.00 (ref)	
Yes	129	9	0.60 (0.28-1.28)	*0.19*	14	0.68 (0.33-1.40)	*0.30*	24	0.78 (0.42-1.45)	*0.44*
Female reproductive organ cancer										
No	1228	132	1.00 (ref)		167	1.00 (ref)		263	1.00 (ref)	
Yes	67	14	1.59 (0.72-3.51)	*0.25*	13	1.56 (0.74-3.27)	*0.24*	17	1.02 (0.52-2.02)	*0.94*
Unknown primary site										
No	1234	139	1.00 (ref)		176	1.00 (ref)		270	1.00 (ref)	
Yes	63	7	0.76 (0.29-1.99)	*0.58*	5	0.54 (0.19-1.54)	*0.25*	12	0.80 (0.38-1.66)	*0.55*

*Abbreviations*: EA, esophageal adenocarcinoma; GCA, gastric cardiac adenocarcinoma; DGA, distal gastric adenocarcinoma; OR, odds ratio; CI, confidence interval.

^a^Results were estimated from multivariate polytomous logistic regression, with adjustment for age, sex, race, education, birth place, cigarette smoking status, body mass index, history of diabetes, and history of other malignancies. History of cancer among first degree relatives was unknown for 17 participants.

^b^Gastrointestinal cancer includes malignant neoplasm of the gastrointestinal tract, including esophagus, stomach, small intestine, colon, rectum, rectosigmoid junction, anus, liver, gallbladder, intrahepatic and extrahepatic bile ducts, pancreas, and other and ill-defined sites within the digestive organs and peritoneum.

We also investigated whether family history of gastroesophageal disorders including any ulcer, gastritis, hiatal hernia, or Barrett’s esophagus was associated with risk of adenocarcinomas at the three sites, with adjustment for matching factors as well as other known risk factors of these adenocarcinomas (Table 3). Results were similar without adjustment for these other known risk factors (Additional file 1: Table S2). Family history of Barrett’s esophagus was rarely reported by the study participants. Risk of EA was positively associated with a family history of ulcers (OR = 1.49; 95% = 0.99-2.25) and a family history of hiatal hernia (OR = 2.04; 95% CI = 1.12-3.71). Risk of GCA was also associated with a family history of hiatal hernia (OR = 2.31; 95% CI = 1.37-3.91). There was no significant association between family history of gastroesophageal disorders and risk of DGA.

**Table 3:** Family history of gastroesophageal disorders and risk of esophageal and gastric adenocarcinoma

**History among first degree relatives**		**EA**			**GCA**			**DGA**		
	**Controls**	**Cases**	**OR (95% CI)**^**a**^	***P***^**a**^	**Cases**	**OR (95% CI)**^**a**^	***P***^**a**^	**Cases**	**OR (95% CI)**^**a**^	***P***^**a**^
Any ulcer										
No	1010	108	1.00 (ref)		138	1.00 (ref)		223	1.00 (ref)	
Yes	279	38	1.49 (0.99-2.25)	0.057	40	1.13 (0.77-1.66)	0.54	59	1.04 (0.74-1.47)	0.80
Gastritis										
No	1168	134	1.00 (ref)		165	1.00 (ref)		257	1.00 (ref)	
Yes	112	12	0.97 (0.51-1.87)	0.93	9	0.59 (0.29-1.21)	0.15	24	1.02 (0.62-1.67)	0.95
Hiatal hernia										
No	1200	130	1.00 (ref)		153	1.00 (ref)		268	1.00 (ref)	
Yes	80	16	2.04 (1.12-3.71)	0.020	22	2.31 (1.37-3.91)	0.002	10	0.90 (0.44-1.84)	0.77
Barrett’s esophagus										
No	1272	141	1.00 (ref)		169	1.00 (ref)		259	1.00 (ref)	
Yes	5	2	-	-	0	-	-	0	-	-

*Abbreviations*: EA, esophageal adenocarcinoma; GCA, gastric cardiac adenocarcinoma; DGA, distal gastric adenocarcinoma; OR, odds ratio; CI, confidence interval.

^a^Results were estimated from multivariate polytomous logistic regression, with adjustment for age, sex, race, education, birth place, cigarette smoking status, body mass index and history of diabetes. History of any ulcer, gastritis, hiatal hernia, and Barrett’s esophagus among first degree relatives was unknown for 28, 42, 44, and 75 participants respectively.

Table 4 presents the combined effects of personal and family history of hiatal hernia on risk of EA, GCA, and DGA. Risk of EA was slightly elevated among individuals with a family history of hiatal hernia but no personal history of hiatal hernia (OR, 1.26; 95% CI, 0.52-3.08), intermediate among those with a personal history of hiatal hernia but no family history of hiatal hernia (OR, 4.91; 95% CI, 3.04-7.93), and highest among individuals with both a personal and a family history of hiatal hernia (OR, 10.75; 95%, 4.26-27.12). Both personal and family histories of hiatal hernia were associated with higher risk of GCA; risk was also highest among individuals with a personal and a family history of hiatal hernia. We also included a product term in the regression model to test for interaction between personal and family histories of hiatal hernia for their effect on risk of EA and GCA, and both interactions were not statistically significant (P = 0.50 and 0.48 respectively). There was no association between personal or family history of hiatal hernia and risk of DGA (Table 4).

**Table 4:** Family and personal history of hiatal hernia and risk of esophageal and gastric adenocarcinoma

**Personal history of hiatal hernia**	**Family history of hiatal hernia**		**EA**			**GCA**			**DGA**		
		**Controls**	**Cases**	**OR (95% CI)**^**a**^	***P***^***a***^	**Cases**	**OR (95% CI)**^**a**^	***P***^***a***^	**Cases**	**OR (95% CI)**^**a**^	***P***^***a***^
No	No	1117	93	1.00 (ref)		129	1.00 (ref)		249	1.00 (ref)	
No	Yes	68	6	1.26 (0.52-3.08)	0.61	13	1.89 (0.99-3.61)	0.056	8	0.89 (0.40-1.97)	0.77
Yes	No	82	36	4.91 (3.04-7.93)	<0.001	23	2.36 (1.40-3.97)	0.001	17	1.44 (0.80-2.60)	0.22
Yes	Yes	12	10	10.75 (4.26-27.12)	<0.001	9	6.55 (2.60-16.50)	<0.001	2	-	-
				*P for interaction=*	*0.50*			*0.48*			*0.99*

*Abbreviations*: EA, esophageal adenocarcinoma; GCA, gastric cardiac adenocarcinoma; DGA, distal gastric adenocarcinoma; OR, odds ratio; CI, confidence interval.

^a^Results were estimated from multivariate polytomous logistic regression, with adjustment for age, sex, race, education, birth place, cigarette smoking status, body mass index and history of diabetes.

## Discussion

In this large population-based case–control study, we found site-specific associations between family history of cancer or gastroesophageal disorders and risk of EA, GCA, and DGA. Family history of cancer in the prostate was associated with an increased risk of EA; family history of esophageal cancer was associated with an increased risk of GCA; and family history of gastrointestinal cancer and particularly gastric cancer was associated with an increased risk of DGA. In addition, family history of hiatal hernia was associated with an increased risk of both EA and GCA, an effect that was more pronounced among individuals with a personal history of hiatal hernia. To our knowledge, this is the first report of an association between family history of hiatal hernia and risk of EA and GCA.

The rapid increase in the incidence of EA over the past few decades in Western countries may indicate a strong contribution of environmental factors to the etiology of EA [21], but genetic factors may also play a role. To date, there is a paucity of genetic association studies of EA [4]. Given that Barrett’s esophagus is an established risk factor for EA [22,23] and Barrett’s esophagus patients have a more than 30 times greater risk of developing EA [21], most previous genetic studies compared patients with Barrett’s esophagus and/or EA with controls and found evidence of familial aggregation of these conditions [4]. It has been suggested that polymorphisms in genes involved in the detoxification of xenobiotics and luminal toxic agents (e.g. GSTM1, GSTT1, GSTP1) as well as those involved in the regulation of cell cycle progression (e.g. CCND1) may play a role in individual susceptibility to EA [4]. Recently, a genome-wide association study of Barrett’s esophagus [24] has identified two susceptibility loci on chromosomes 16q24 and 6p21. The closest protein-coding gene to the 16q24 locus is FOXF1, a gene implicated in esophageal development and structure. This finding suggests that structural factors related to the development of the esophagus may play a role in the etiology of Barrett’s esophagus. It is consistent with the fact that most of individuals affected by Barrett’s esophagus have a history of hiatal hernia in their lower esophagus. We found that EA risk was higher among individuals with a family history of hiatal hernia (OR = 2.05) than those without this history and that risk is highest among those with both a personal and a family history of hiatal hernia (OR = 10.75). The observation that EA risk increases with an increasing number of family members (participant’s family plus participant him/herself) affected by hiatal hernia suggests that genetic factors that are involved in the development of hiatal hernia and Barrett’s esophagus play a role in the development of EA. In addition, even though in our study personal and family history of hiatal hernia was obtained though self-report, our results are consistent with the strong evidence of familial aggregation of Barrett’s esophagus and EA from previous studies [4,25,26], in which patients were ascertained through clinical diagnosis.

We also found an increased risk of EA among individuals with a family history of prostate cancer but lower risk among those with a family history of breast cancer and these associations did not substantially change after controlling for gender-specific sibship size. Such results were unexpected and require confirmation. Reports of associations between family history of breast or prostate cancer and risk of gastroesophageal cancer are sparse and have been inconsistent [11,12,14]. A multicenter, population-based case–control study conducted in the U.S. during 1993-1995 [12] found that family history of breast cancer was associated with increased risks of EA and non-cardia gastric adenocarcinoma, while the association of family history of prostate cancer with lower risk of these two types of cancers was not statistically significant. In a case–control study of patients seen at the Memorial Sloan-Kettering Cancer Center from 1992 to 1994, family history of breast or prostate cancer was not associated with an increased risk of EA and GCA [11]. In a study based on the Swedish Family-Cancer Database [14], maternal breast cancer was associated with an increased risk of gastric cancer in the offspring (standardized incidence ratio = 1.84, 95% CI = 1.02-3.04). Despite these sparse observations, our results are consistent with the strong male dominance in EA [27]. Among white Americans, males have 7.7 times higher risk of getting EA than females [28]. In addition, this gender difference cannot be adequately explained by differences in known risk factors for EA including gastroesphageal reflux diseases, obesity, and tobacco consumption [29], suggesting that other unknown factors such as sex hormonal factors and related signaling axes may play important roles in the development of EA [27,29]. Experimental studies have shown that estrogen may have an inhibitory effect [27] and androgens may have growth-enhancing effects [30] on the carcinogenic process of the esophagus. Compared to individuals without a family history of prostate cancer, those with a positive family history may have a higher exposure to androgens [31] or enhanced susceptibility to the effect of androgens, leading to a higher risk of esophageal cancer. Similarly, individuals with a family history of breast cancer may have higher exposure to estrogen [32] or enhanced susceptibility to the effect of estrogen, therefore resulting in a reduced risk of esophageal cancer.

EA and GCA display some similar descriptive epidemiological features [1] and risk factors. Low intake of fiber[19], hiatal hernia, and Barrett’s esophagus [17,22,23] all have been associated with increased risks of both EA and GCA. Therefore, it is not surprising that family history of esophageal cancer was associated with an increased risk of GCA, which might be explained by common family dietary habits such as low fiber intake, or by a shared genetic susceptibility to hiatal hernia and Barrett’s esophagus. In the current study, additional adjustment for fiber intake did not explain the association between family history of esophageal cancer and risk of GCA, suggesting that shared genetic susceptibility may play a more important role.

The observed association between family history of a gastrointestinal cancer or of gastric cancer and an increased risk of DGA could be explained by shared susceptibility to both genetic and environmental factors. Family studies [33,34] have shown that shared and non-shared environmental factors largely accounted for the variation in gastric cancer whereas genetic factors accounted for only a small proportion of gastric cancer susceptibility. This observation is consistent with the sharp decline in gastric cancer incidence over the past 40 years, owing to improvements in diet and food storage methods as well as a decline in the prevalence of *H. pylori* infection [35]. Common exposure to *H. pylori* may explain the positive associations of GCA risk with family history of gastric cancer and family history of other gastrointestinal cancers, as *H. pylori* infection has been shown to increase the risk of not only gastric cancer, but also pancreatic cancer [36]. Data on history of *H. pylori* infection was not available for majority of our study participants; therefore we were unable to investigate whether history of *H. pylori* infection was a potential effect modifier of the association between family history of gastric cancer and risk of GCA. However, the associations between family history of these gastrointestinal cancers and increased risk of GCA may also be mediated by shared genetic susceptibilities [37]. For example, candidate gene studies have consistently found that polymorphisms in the IL-1β and MTHFR genes were associated with individual susceptibility to both intestinal-type gastric cancer and pancreatic cancer [4,38,39].

The strengths of this study include the population-based design, relatively large sample size, separation of gastroesophageal adenocarcinoma by anatomical sites, and data on family history of cancer and non-malignant gastroesophageal disorders. Results from our study are compatible with the limited evidence to date and known etiological mechanisms of EA, GCA, and DGA. Our study also has a number of limitations. First, even though we excluded NOK interview data from our analyses, our data on family history were reported by study participants rather than by family member themselves; therefore they may be subject to recall bias, under-ascertainment of family history, and misclassification of the primary tumor site. The lack of excessive reporting of family history of esophageal cancer by cases with EA indicates that our results were most likely not affected by selective recall bias. In addition, misclassification of the primary tumor site of a relative was lessened by only collecting data on first-degree relatives [40]. Nevertheless, it is not possible to collect information on histology (e.g. adenocarcinoma vs. squamous cell carcinoma) or specific tumor subsite (gastric cardia vs. non-cardia) among those who reported esophageal/gastric cancers in first-degree relatives. Second, our sample size was insufficient to evaluate very rare cancers in family members, to examine risk patterns separately for parents vs. siblings or to assess whether the number of affected relatives refined risk estimates. In particular, there was only one EA patient and seven controls with a positive family history of EA, our study was not sufficient powered to examine the association between family history of EA and risk of EA. However, sparse-data bias was unlikely to have occurred, because: a) reducing over-stratification in our regression analyses, i.e. removing additional adjustment for known risk factors (Additional file 1: Tables S1 and S2 versus Tables 2 and 3), did not change our results substantially; and b) our results were very similar with and without Firth’s correction [41] for sparse data (data not shown). A pooled analysis of data from all relevant population-based studies is needed for these additional analyses. Third, we recognize that if the effect of family history of cancer or gastroesophageal disorders may be exerted through common family dietary habits or by a shared genetic susceptibility, adjusting for known risk factors for gastroesophageal cancer may underestimate the actual strength of the association between family history of cancer/gastroesophageal disorders and risk of gastroesophageal cancer. However, overadjustment is unlikely because, as mentioned above, removing additional adjustment for known risk factors did not change our results substantially. Fourth, BMI was categorized using sex-specific quartiles rather than the World Health Organization classification to avoid sparse data for some categories. In addition, we did not control for history of *Helicobacter pylori (H. pylori)* infection in multivariate analyses, because serum IgG antibodies to H. pylori whole-cell antigens (Helico-G) and CagA were not measured for majority of study participants [16]. Furthermore, we were unable to distinguish shared genetics from shared early-life environmental exposures among first-degree relatives, as environmental exposure histories of participants’ relatives were not available.

## Conclusions

In summary, our data suggest that family history of cancers at specific sites may be associated with risk of EA, GCA, and DGA. These associations may be mediated by shared environmental factors as well as by genetic factors. Our limited sample size warrants cautious interpretation of the observed null associations. Pooled analyses of individual studies are required for more precise quantification and more thorough investigation of the associations of family history of cancer or gastroesophageal disorders with the development of EA, GCA, and DGA.

## Abbreviations

BMI: Body mass index; CI: Confidence interval; DGA: Distal gastric adenocarcinoma; EA: Esophageal adenocarcinoma; GCA: Gastric cardiac adenocarcinoma; ICD-9: The international classification of diseases 9^th^ version code; ICD-O: The international classification of disease for oncology code; NOK: Next of kin; OR: Odds ratio; SEER: Surveillance, epidemiology, and end results program.

## Competing interests

All authors declare that they have no competing interests.

## Authors’ contributions

XJ analyzed data, interpreted results, and drafted the manuscript; CCT provided data management support; LB was involved in obtaining funding, data acquisition, and providing critical revision of the manuscript, and AHW was involved in obtaining funding, data collection, and initiated and supervised analysis of this paper. All authors read and approved the final manuscript.

## Pre-publication history

The pre-publication history for this paper can be accessed here:

http://www.biomedcentral.com/1471-2407/14/60/prepub

## Supplementary Material

Additional file 1: Table S1 Family history of cancer and risk of esophageal and gastric adenocarcinoma adjusted for matching factors only. **Table S2.** Family history of gastroesophageal disorders and risk of esophageal and gastric adenocarcinoma adjusted for matching factors only. Click here for file
