# Supplementary material for: Family history of cancer and gastroesophageal disorders and risk of esophageal and gastric adenocarcinomas: a case–control study
Source: BMC Cancer. 2014 Feb 4;14:60. doi: 10.1186/1471-2407-14-60 (PMC3915076; doi:10.1186/1471-2407-14-60)
Supplement: Additional file 1: Table S1 — Family history of cancer and risk of esophageal and gastric adenocarcinoma adjusted for matching factors only. Table S2. Family history of gastroesophageal disorders and risk of esophageal and gastric adenocarcinoma adjusted for matching factors only. [file 1471-2407-14-60-S1.docx]

Table S1. Family history of cancer and risk of esophageal and gastric adenocarcinoma adjusted for matching factors only.

| History of cancer among |  | EA | | |  | GCA | | |  | DGA | | |
| --- | --- | --- | --- | --- | --- | --- | --- | --- | --- | --- | --- | --- |
| first degree relatives | Controls | Cases | OR (95% CI)^a^ | *P^a^* |  | Cases | OR (95% CI)^a^ | *P^a^* |  | Cases | OR (95% CI)^a^ | *P^a^* |
| Any cancer |  |  |  |  |  |  |  |  |  |  |  |  |
| No | 668 | 68 | 1.00 (ref) |  |  | 90 | 1.00 (ref) |  |  | 160 | 1.00 (ref) |  |
| Yes | 629 | 78 | 1.14 (0.80-1.62) | 0.47 |  | 91 | 0.99 (0.72-1.36) | 0.96 |  | 122 | 0.87 (0.66-1.14) | 0.31 |
| Late-onset | 481 | 58 | 1.05 (0.72-1.54) | 0.79 |  | 63 | 0.86 (0.61-1.23) | 0.41 |  | 89 | 0.86 (0.64-1.16) | 0.32 |
| Early-onset | 148 | 20 | 1.46 (0.85-2.50) | 0.17 |  | 28 | 1.45 (0.91-2.32) | 0.12 |  | 33 | 0.90 (0.58-1.39) | 0.63 |
| ***Gastrointestinal cancer*** |  |  |  |  |  |  |  |  |  |  |  |  |
| Any gastrointestinal cancer^2^ |  |  |  |  |  |  |  |  |  |  |  |  |
| No | 1101 | 125 | 1.00 (ref) |  |  | 148 | 1.00 (ref) |  |  | 226 | 1.00 (ref) |  |
| Yes | 196 | 21 | 0.93 (0.57-1.53) | 0.77 |  | 33 | 1.20 (0.79-1.81) | 0.40 |  | 56 | 1.33 (0.94-1.89) | 0.11 |
| Late-onset | 153 | 16 | 0.88 (0.50-1.53) | 0.64 |  | 26 | 1.18 (0.75-1.87) | 0.47 |  | 40 | 1.23 (0.83-1.84) | 0.30 |
| Early-onset | 43 | 5 | 1.14 (0.44-2.97) | 0.79 |  | 7 | 1.24 (0.55-2.84) | 0.61 |  | 16 | 1.64 (0.88-3.05) | 0.12 |
| Esophageal cancer |  |  |  |  |  |  |  |  |  |  |  |  |
| No | 1290 | 145 | 1.00 (ref) |  |  | 176 | 1.00 (ref) |  |  | 280 | 1.00 (ref) |  |
| Yes | 7 | 1 | - | *-* |  | 5 | 4.83 (1.49-15.65) | 0.009 |  | 2 | - | *-* |
| Gastric cancer |  |  |  |  |  |  |  |  |  |  |  |  |
| No | 1241 | 141 | 1.00 (ref) |  |  | 171 | 1.00 (ref) |  |  | 260 | 1.00 (ref) |  |
| Yes | 56 | 5 | 0.82 (0.32-2.10) | 0.67 |  | 10 | 1.33 (0.66-2.68) | 0.43 |  | 22 | 1.56 (0.91-2.66) | 0.10 |
| Colorectal cancer |  |  |  |  |  |  |  |  |  |  |  |  |
| No | 1222 | 138 | 1.00 (ref) |  |  | 171 | 1.00 (ref) |  |  | 260 | 1.00 (ref) |  |
| Yes | 75 | 8 | 0.98 (0.46-2.11) | 0.96 |  | 10 | 0.93 (0.47-1.85) | 0.83 |  | 22 | 1.42 (0.84-2.40) | 0.20 |
| Liver cancer |  |  |  |  |  |  |  |  |  |  |  |  |
| No | 1267 | 139 | 1.00 (ref) |  |  | 179 | 1.00 (ref) |  |  | 276 | 1.00 (ref) |  |
| Yes | 30 | 7 | 1.91 (0.81-4.53) | 0.14 |  | 2 | - | *-* |  | 6 | 0.94 (0.37-2.38) | 0.89 |
| Pancreatic cancer |  |  |  |  |  |  |  |  |  |  |  |  |
| No | 1275 | 145 | 1.00 (ref) |  |  | 176 | 1.00 (ref) |  |  | 276 | 1.00 (ref) |  |
| Yes | 22 | 1 | - | *-* |  | 5 | 1.36 (0.50-3.67) | 0.55 |  | 6 | 1.25 (0.47-3.36) | 0.66 |
| ***Non-gastrointestinal cancer*** |  |  |  |  |  |  |  |  |  |  |  |  |
| Lung cancer |  |  |  |  |  |  |  |  |  |  |  |  |
| No | 1202 | 132 | 1.00 (ref) |  |  | 164 | 1.00 (ref) |  |  | 267 | 1.00 (ref) |  |
| Yes | 95 | 14 | 1.15 (0.63-2.10) | 0.64 |  | 17 | 1.14 (0.66-1.98) | 0.63 |  | 15 | 0.92 (0.51-1.65) | 0.78 |
| Upper respiratory organ cancer |  |  |  |  |  |  |  |  |  |  |  |  |
| No | 1172 | 128 | 1.00 (ref) |  |  | 157 | 1.00 (ref) |  |  | 260 | 1.00 (ref) |  |
| Yes | 125 | 18 | 1.16 (0.68-1.98) | 0.59 |  | 24 | 1.28 (0.80-2.05) | 0.31 |  | 22 | 0.94 (0.57-1.54) | 0.80 |
| Skin/bone/connective tissue cancer |  |  |  |  |  |  |  |  |  |  |  |  |
| No | 1225 | 141 | 1.00 (ref) |  |  | 174 | 1.00 (ref) |  |  | 272 | 1.00 (ref) |  |
| Yes | 72 | 5 | 0.51 (0.20-1.30) | 0.16 |  | 7 | 0.58 (0.26-1.29) | 0.18 |  | 10 | 1.00 (0.50-2.03) | 0.99 |
| Lymphatic/hematopoietic cancer |  |  |  |  |  |  |  |  |  |  |  |  |
| No | 1256 | 141 | 1.00 (ref) |  |  | 179 | 1.00 (ref) |  |  | 272 | 1.00 (ref) |  |
| Yes | 41 | 5 | 0.92 (0.36-2.40) | 0.87 |  | 2 | - | *-* |  | 10 | 1.24 (0.59-2.60) | 0.57 |
| Prostate cancer |  |  |  |  |  |  |  |  |  |  |  |  |
| No | 1224 | 130 | 1.00 (ref) |  |  | 170 | 1.00 (ref) |  |  | 272 | 1.00 (ref) |  |
| Yes | 73 | 16 | 2.03 (1.13-3.64) | 0.017 |  | 11 | 1.05 (0.54-2.03) | 0.88 |  | 10 | 0.65 (0.32-1.30) | 0.22 |
| Breast cancer |  |  |  |  |  |  |  |  |  |  |  |  |
| No | 1168 | 137 | 1.00 (ref) |  |  | 167 | 1.00 (ref) |  |  | 258 | 1.00 (ref) |  |
| Yes | 129 | 9 | 0.54 (0.27-1.10) | 0.087 |  | 14 | 0.71 (0.40-1.27) | 0.25 |  | 24 | 0.92 (0.57-1.47) | 0.72 |
| Female reproductive organ cancer |  |  |  |  |  |  |  |  |  |  |  |  |
| No | 1228 | 132 | 1.00 (ref) |  |  | 167 | 1.00 (ref) |  |  | 263 | 1.00 (ref) |  |
| Yes | 67 | 14 | 2.06 (1.11-3.82) | 0.022 |  | 13 | 1.51 (0.81-2.81) | 0.20 |  | 17 | 1.15 (0.65-2.03) | 0.64 |
| Unknown primary site |  |  |  |  |  |  |  |  |  |  |  |  |
| No | 1234 | 139 | 1.00 (ref) |  |  | 176 | 1.00 (ref) |  |  | 270 | 1.00 (ref) |  |
| Yes | 63 | 7 | 0.98 (0.44-2.22) | 0.97 |  | 5 | 0.57 (0.22-1.44) | 0.23 |  | 12 | 0.75 (0.39-1.44) | 0.38 |

Abbreviations: EA, esophageal adenocarcinoma; GCA, gastric cardiac adenocarcinoma; DGA, distal gastric adenocarcinoma; OR, odds ratio; CI, confidence interval.

^a^Results were estimated from multivariate polytomous logistic regression, with adjustment for age, sex, and race.

Table S2. Family history of reflux conditions and risk of esophageal and gastric adenocarcinoma adjusted for matching factors only.

| History among first degree relatives |  | EA | | |  | GCA | | |  | DGA | | |
| --- | --- | --- | --- | --- | --- | --- | --- | --- | --- | --- | --- | --- |
|  | Controls | Cases | OR (95% CI)^a^ | *P*^a^ |  | Cases | OR (95% CI)^a^ | *P*^a^ |  | Cases | OR (95% CI)^a^ | *P*^a^ |
| Any ulcer |  |  |  |  |  |  |  |  |  |  |  |  |
| No | 1010 | 108 | 1.00 (ref) |  |  | 138 | 1.00 (ref) |  |  | 223 | 1.00 (ref) |  |
| Yes | 279 | 38 | 1.44 (0.96-2.16) | 0.075 |  | 40 | 1.12 (0.76-1.64) | 0.56 |  | 59 | 0.97 (0.70-1.36) | 0.88 |
| Gastritis |  |  |  |  |  |  |  |  |  |  |  |  |
| No | 1168 | 134 | 1.00 (ref) |  |  | 165 | 1.00 (ref) |  |  | 257 | 1.00 (ref) |  |
| Yes | 112 | 12 | 1.06 (0.56-2.01) | 0.86 |  | 9 | 0.63 (0.31-1.28) | 0.20 |  | 24 | 1.04 (0.64-1.69) | 0.87 |
| Hiatal hernia |  |  |  |  |  |  |  |  |  |  |  |  |
| No | 1200 | 130 | 1.00 (ref) |  |  | 153 | 1.00 (ref) |  |  | 268 | 1.00 (ref) |  |
| Yes | 80 | 16 | 1.89 (1.06-3.40) | 0.032 |  | 22 | 2.05 (1.23-3.43) | 0.006 |  | 10 | 0.68 (0.34-1.38) | 0.29 |
| Barrett's esophagus |  |  |  |  |  |  |  |  |  |  |  |  |
| No | 1272 | 141 | 1.00 (ref) |  |  | 169 | 1.00 (ref) |  |  | 259 | 1.00 (ref) |  |
| Yes | 5 | 2 | - | *-* |  | 0 | - | - |  | 0 | - | - |

Abbreviations: EA, esophageal adenocarcinoma; GCA, gastric cardiac adenocarcinoma; DGA, distal gastric adenocarcinoma; OR, odds ratio; CI, confidence interval.

^a^Results were estimated from multivariate polytomous logistic regression, with adjustment for age, sex, and race.
